# Polyethylene glycol-grafted graphene oxide nanosheets in tailoring the structure and reverse osmosis performance of thin film composite membrane

**DOI:** 10.1038/s41598-023-44129-z

**Published:** 2023-10-07

**Authors:** Zahra Sanei, Taranom Ghanbari, Alireza Sharif

**Affiliations:** Polymer Reaction Engineering Department, Faculty of Chemical Engineering, Tarbiat Modares University, P.O. Box 14155-143, Tehran, Iran

**Keywords:** Polymers, Chemical engineering

## Abstract

Introducing hydrophilic polymers such as polyethylene glycol (PEG) within the polyamide (PA) layer of thin film composite (TFC) membranes helps achieve high water desalination performance. Here, PEGs of different molecular weights (X: 1500, 6000, 16,000 g/mol) are effectively introduced into the PA layer of TFC membranes utilizing PEG-grafted graphene oxide (GOP_X_) nanosheets and their effects on the physicochemical properties and reverse osmosis (RO) performance of the thin film nanocomposite (TFN) membranes are investigated. Among the TFNs prepared the GOP_16000_/TFN exhibits the best performance with 68% improvement in water flux and almost constant salt rejection compared to those of the bare TFC. The influence of PEG molecular weight on the RO performance of the membranes is interpreted by different surface and bulk hydrophilicity as well as thickness and surface roughness of PA layers of GOP_X_/TFNs. Furthermore, TFNs with thinner and smoother PA layers and thus higher water flux are obtained by dispersing GOP_X_s in the aqueous phase of the PA interfacial polymerization reaction than by dispersing them in the organic phase of the reaction. Finally, the high antifouling potential of TFNs containing PEG-grafted GOs is demonstrated.

## Introduction

Polyamide thin-film nanocomposite (PA-TFN) membranes made by incorporating nanoparticles (NPs) into the PA thin layer are promising strategies for wastewater treatment and desalination^1^. The right choice of NPs with desired surface modification would ensure the fabricating of fouling-resistant TFNs possessing high water flux and salt rejection in different membrane processes, especially in reverse osmosis (RO)^2,3^. Among the recommended NPs for PA-TFN membranes preparation in the literature, graphene oxide (GO) nanosheets have shown remarkable efficacy in resolving the permeability-selectivity trade-off and membrane fouling issues^4^. In particular, the hydrophilic nature of GO allows water molecules to be embedded in its interlayer structure, thereby providing efficient transport pathways for water molecules across the polyamide (PA) layer^5,6^.

Furthermore, the inherent surface functional groups of GO such as hydroxyl and carboxylic acid provide suitable reaction sites for covalent modifications preventing nonspecific interactions among GOs, promoting their dispersion in the PA layer and introducing target functional groups to improve the TFN separation properties^6,7^. For instance, hydrophilic chitosan polysaccharide was grafted onto graphene oxide and the modified nanosheets were introduced into the top layer of a PA-TFC membrane. The resultant TFN membrane became more hydrophilic and smoother than the TFC membrane exhibiting high water flux and salt rejection^5^. Moreover, Zhang et al.^6^ prepared TFN membranes by incorporating nanofillers of p-aminophenol-modified graphene oxide (mGO) into the PA layer during the interfacial polymerization reaction. The addition of mGO into the active layer reduced the layer thickness and water contact angle by 79.1% and 30.7%, respectively, thereby displaying 24.5% and 99.7% increases in water flux and NaCl rejection, respectively. β-cyclodextrin functionalized graphene oxide nanosheets (β-CD-f-GO) were also used to fabricate TFN membranes possessing improved reverse osmosis and antifouling properties. These enhanced performances were ascribed to the role of the modified nanosheets in increasing the hydrophilicity and decreasing the surface roughness of the top PA layer^7^. Recently, Mahdavi and Rahimi^8^ synthesized TFN membranes containing GO nanoparticles modified with poly(2-(methacryloyloxy)ethyl dimethyl-(3-sulfopropyl)ammonium hydroxide) zwitterionic polymer in the top PA layer. The water flux and antifouling properties of the membranes were greatly improved while the salt rejection remained unchanged relative to the bare TFC membrane. In addition, Ma et al.^9^ incorporated GO functionalized with (poly(sulfobetaine methacrylate)) in the active PA layer of a TFC membrane and reported a twofold increase in the water flux while 12% and 5% compromise in the rejection of NaCl (monovalent ions) and MgSO_4_ (divalent ions), respectively.

Poly (ethylene glycol) (PEG) is a hydrophilic polymer widely used as an additive for morphology control and increasing the water flux and fouling resistance of single-layer water desalination membranes and support layers of TFCs^10^. In recent years, however, the grafting of PEG derivatives on the thin PA layer of TFC-RO membranes has been reported by some authors^11–16^. For example, Van Wegner et al.^12^ grafted poly (ethylene glycol) di-glycidyl ether (PEGDE) onto the surface of a RO membrane. Although the modified membranes with PEGDE generally exhibited promoted fouling resistance against charged surfactants and emulsions, their water flux was decreased due to this surface modification. Kang et al.^13^ utilized a chemical reaction between unreacted acyl halide groups on the PA surface of a TFC membrane and amino groups of aminopolyethylene glycol monomethyl ether (MPEG-NH_2_) to introduce poly(ethylene glycol) chains on the membrane active layer and the modified membrane sowed enhanced surface hydrophilicity and thus improved organic fouling resistance. The same group reported elsewhere that grafting PEGDE on the top surface of TFC-RO membranes renders fouling-resistant membranes against cationic surfactants and proteins^14^.

Photo-induced graft polymerization and plasma polymerization have also been used to incorporate different PEG-based moieties on the active layer of TFC membranes to develop membranes with higher fouling resistance and separation properties^15,16^. Nevertheless, the direct grafting of PEG-based polymers onto the membrane surface would find limited industrial applications due to the need for multi-step reactions under harsh conditions. Furthermore, most of the TFC membranes having top surfaces grafted with polymers suffer from declined permeance as a result of the direct grafting approach^17^. On the other hand, the embedding of hydrophilic PEG chains, during the IP reaction, within the top PA layer of TFC membranes may facilitate water transport across the layer and improve the membrane flux. To achieve this goal, immobilizing PEG chains on a substrate, e.g. the GO substrate, reduces the risk of leaching these highly hydrophilic chains during the interfacial polymerization reaction and water purification process. Furthermore, utilizing this strategy will be useful to improve the dispersion of GO nanosheets in the top PA layer of the membranes.

Herein, we utilized GO nanosheets as substrates for PEG chains in the top layers of PA-TFC membranes to exploit both the advantages of nanosheets and hydrophilic polymer for water desalination purposes, simultaneously. Furthermore, to study the effect of the molecular weight (MW) of PEG on the RO performance of TFC membranes, PEG chains with different MWs, including 1500, 6000 and16000 g/mol have been grafted onto GO nanosheets and introduced to the top layers of the PA-TFC membranes through the aqueous or organic phases of the interfacial polymerization reaction during the preparation of the membranes. Thus, the modified nanoparticles were successfully concentrated on the top layer of TFN membranes during the interfacial polymerization process. The results reveal the determining role of the MW of the grafted PEG chains on the morphology and separation performance of TFN membranes in an RO process.

## Experimental

### Materials

Graphite (99.55%, particle size < 50 µm), sulfuric acid (H_2_SO_4_, 98%), potassium permanganate (KMnO_4_), dimethylformamide (DMF, 99.8%), polyethylene glycols (PEG, M_w_ = 1500, 6000, 16,000 g/mol), tetrahydrofuran (THF), triethylamine (TEA), sodium carbonate (Na_2_CO_3_), sodium hydroxide (NaOH) and sodium dodecyl sulfate (SDS, > 99%) were purchased from Merck. Thionyl chloride (SOCl_2_) was purchased from CDH, India. Hydrogen peroxide (H_2_O_2_, 35%), hydrochloric acid (HCl, 37%) and n-hexane were obtained from Dr. Mojallali CO, Iran. Ethanol (C_2_H_5_OH, 96%) was obtained from Taghtirkhorasan CO., Iran. Polysulfone microporous sublayer reinforced with a polyester non-woven support with a molecular weight cut-off (MWCO) of 5400 Da, was supplied by the Sharif Membrane Technology Center (SMTC), Iran. Trimesoyl chloride (TMC), and m-phenylenediamine (MPD) were obtained from Sigma Aldrich.

### Synthesis of GO-PEG nanosheets

**Figure 1:**
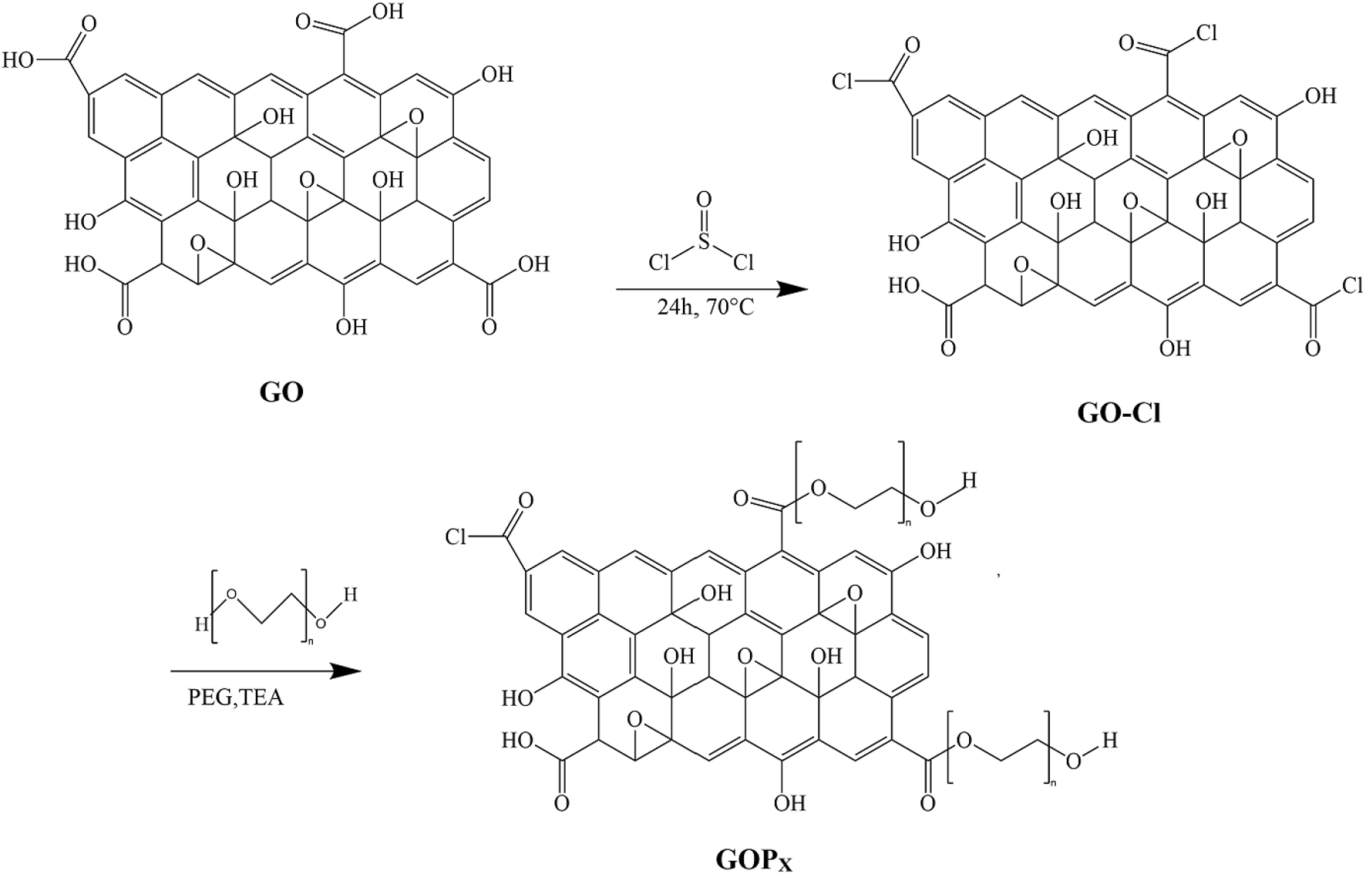
Chemical routes to the synthesis of GOP_X_s.

GO nanosheets were synthesized from graphite powders by modified Hummers’ method as reported in our previous work^18^. For the GO chlorination, first, 200 mg of the nanosheets were added to 10 mL DMF under ultrasonication to obtain a stable DMF/GO suspension. Then, 40 mL of SOCl_2_ was added to the suspension followed by refluxing for 24 h at 70 °C. Finally, the mixture was washed five times by centrifuging to remove excess SOCl_2_ and the chlorinated GO nanosheets (GO-Cl) were collected. Subsequently, 2 g of PEG of a certain MW and 5 mL of TEA were added to the chlorinated GOs and the mixtures were stirred for 3 days at 120 °C^19^. The final suspensions were then poured into ethanol and dried at 60 °C in a vacuum oven to obtain various PEGylated GOs, designated as GOP_X_, where X: 1, 2 and 3 refer to PEGs with MWs of 1500, 6000 and 16,000 g/mol, respectively. Figure 1 represents the chemical reactions for the preparation of GOP_X_s.

### Preparation of TFN membranes

TFN membranes were prepared by in situ IP reaction of 2 wt% MPD in water with 0.2 wt% TMC in n-hexane atop the polysulfone substrate. The aqueous phase containing 2 wt% Na_2_CO_3_, 0.5 wt% SDS and 0.004 wt% GO or each GOP_X_ was ultrasonicated for 1 h to obtain a stable suspension before the IP reaction. The aqueous suspension was poured on the polysulfone substrate for 2 min and then the substrate was held vertically to remove the excess MPD. The MPD-impregnated substrate was contacted with the organic phase containing TMC for 2 min and a PA thin film was formed on the substrate. Finally, the membranes were annealed in an oven for 10 min at 80 °C to complete the IP reaction and kept in deionized water before testing^20^. To investigate the effect of dispersing the nanosheets in the organic phase, instead of the aqueous phase, on the morphology and final performance of the membranes, GOP_3_ was also added in the organic phase for the IP reaction as explained above. The prepared membranes are designated according to Table 1.

**Table 1:** The designation of the prepared membranes.

Membrane	Nanosheet	IP Phase in which nanosheets were dispersed
TFC	–	–
GO/TFN	GO	Aqueous
GOP_1_/TFN	GO-PEG1500	Aqueous
GOP_2_/TFN	GO-PEG6000	Aqueous
GOP_3_/TFN	GO-PEG16000	Aqueous
GOP_3,Org_/TFN	GO-PEG16000	Organic

### Characterization

X-ray diffraction (XRD) patterns of the nanosheets were obtained by Philips diffractometer with Cu-Kα radiation (λ = 1. 45 Å) at 40 kV/30 mA in the scanning range 2θ of 5° to 60°. Thermogravimetric analysis (TGA) was performed on an STA504 instrument (Bahr, Germany) under an argon atmosphere in the temperature range of 50 °C to 650 °C with a heating rate of 10 $^\circ$C/min. Modified nanoparticles were characterized by Fourier-transform infrared spectroscopy (FTIR, Perkin Elmer spectrum version 10.03.06, USA). The polyamide layer of the membranes was studied by attenuated total reflection Fourier-transform infrared spectroscopy (ATR-FTIR, Perkin Elmer spectrum version 10.03.06, USA) over a wave number range of 800–4000 cm^−1^. The surface topography of the membranes was examined by an atomic force microscope (AFM, Agilent Technologies, Inc., Santa Clara, CA), in a tapping mode. A field-emission scanning electron microscope (FE-SEM, Philips XL-30) was utilized to investigate the membrane’s morphology. The hydrophilicity of the membrane surfaces was characterized by water contact angle measurements using a digital microscope (WCA, Dino-lite instrument) at room temperature.

### Membrane performance assessment

The water desalination performance of the membranes in the RO process was measured by a crossflow filtration device equipped with a circular cell with an effective area of 12.56 cm^2^. The measurements were performed using 2000 ppm NaCl solution at 16 bar pressure. The feed flow rate was adjusted to 70 L/h. The retentate was returned to the feed reservoir by a circulation system during the separation tests.

The permeate flux (F) was obtained by Eq. (1):

1 $$ {\text{J }} = {\text{ V }}/{\text{ A }} \times {\text{ T}} $$

where V is the permeate volume, t is the sample collection time and A is the effective membrane area.

Furthermore, the salt rejection (R) was calculated by Eq. (2):

2 $$ R(\% ) = \left[ {1 - \frac{{C_{p} }}{{C_{f} }}} \right] \times 100 $$

where C_f_ and C_p_ are the concentrations of NaCl in the feed and permeate, respectively^20^. The C_f_ and C_p_ values were determined by a digital conductivity meter (Oakton CON 110).

## Results and discussion

### Characterization of the synthesized GOP_X_s

**Figure 2:**
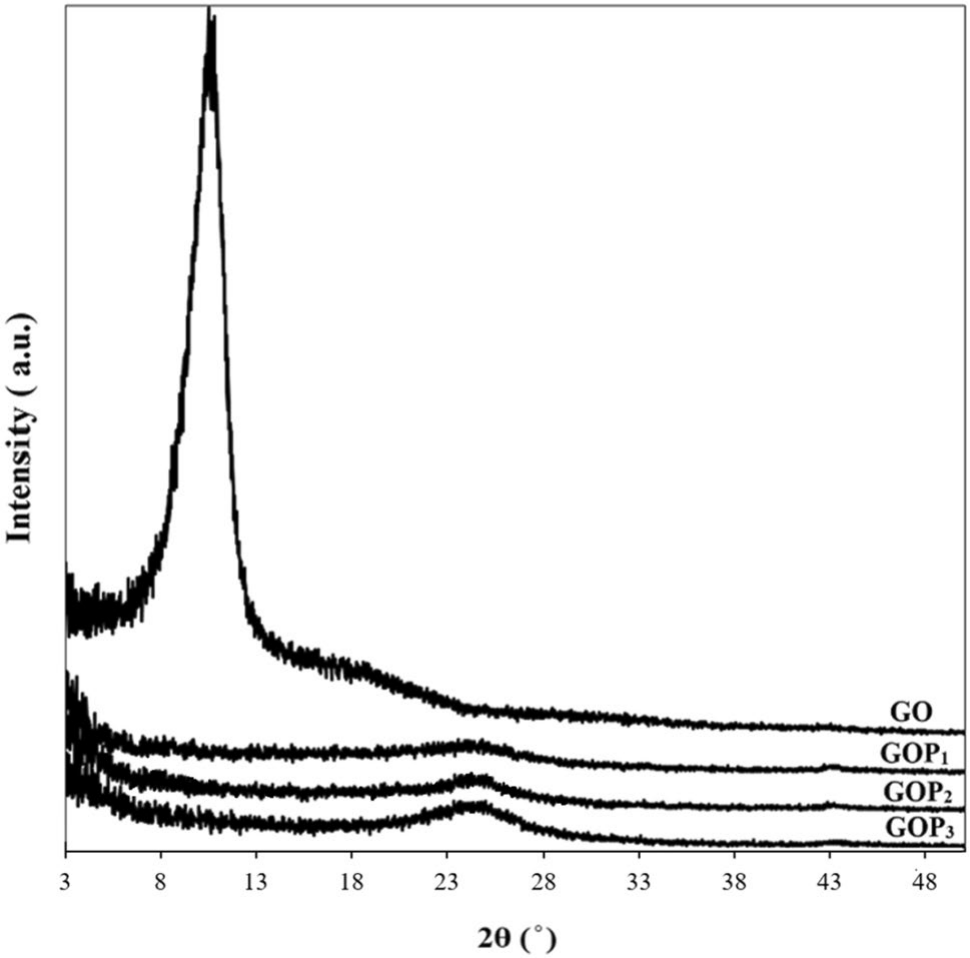
XRD patterns of GO and GOP_X_s.

The XRD patterns of GO and PEGylated GOs are shown in Fig. 2. In the pristine GO pattern, the appearance of a peak at 2$\theta =10.8^\circ$ with a d-spacing of 0.83 nm is attributed to the reflection of the (001) plane^21^. and points to the oxygenated functional groups of GO. The peak at $2\theta$ = 10.8$^\circ$ is disappeared in the XRD patterns of GOP_X_s and the modified nanosheets show additional diffuse peaks at 2$\theta =$ 24$^\circ$. The disappearance of the XRD peak at 2θ = 10.8$^\circ$ can be ascribed to the disordering and increase in interlayer spacing between GO nanosheets due to the grafting of PEG chains. Furthermore, the weak and broad diffraction peaks at 2$\theta =$ 24$^\circ$ in the GOPxs patterns are related to the amorphous structures of PEG chains^22^ because the XRD pattern of the crystalline PEG is characterized by two sharp and strong 2θ peaks at 19.1° and 23.6°, which are assigned to the typical planes of (120) and (112) of PEG^23,24^. These observations confirm the amorphous disordered state and random direction of the graphene oxide layers as a result of the grafting process^25^.

**Figure 3:**
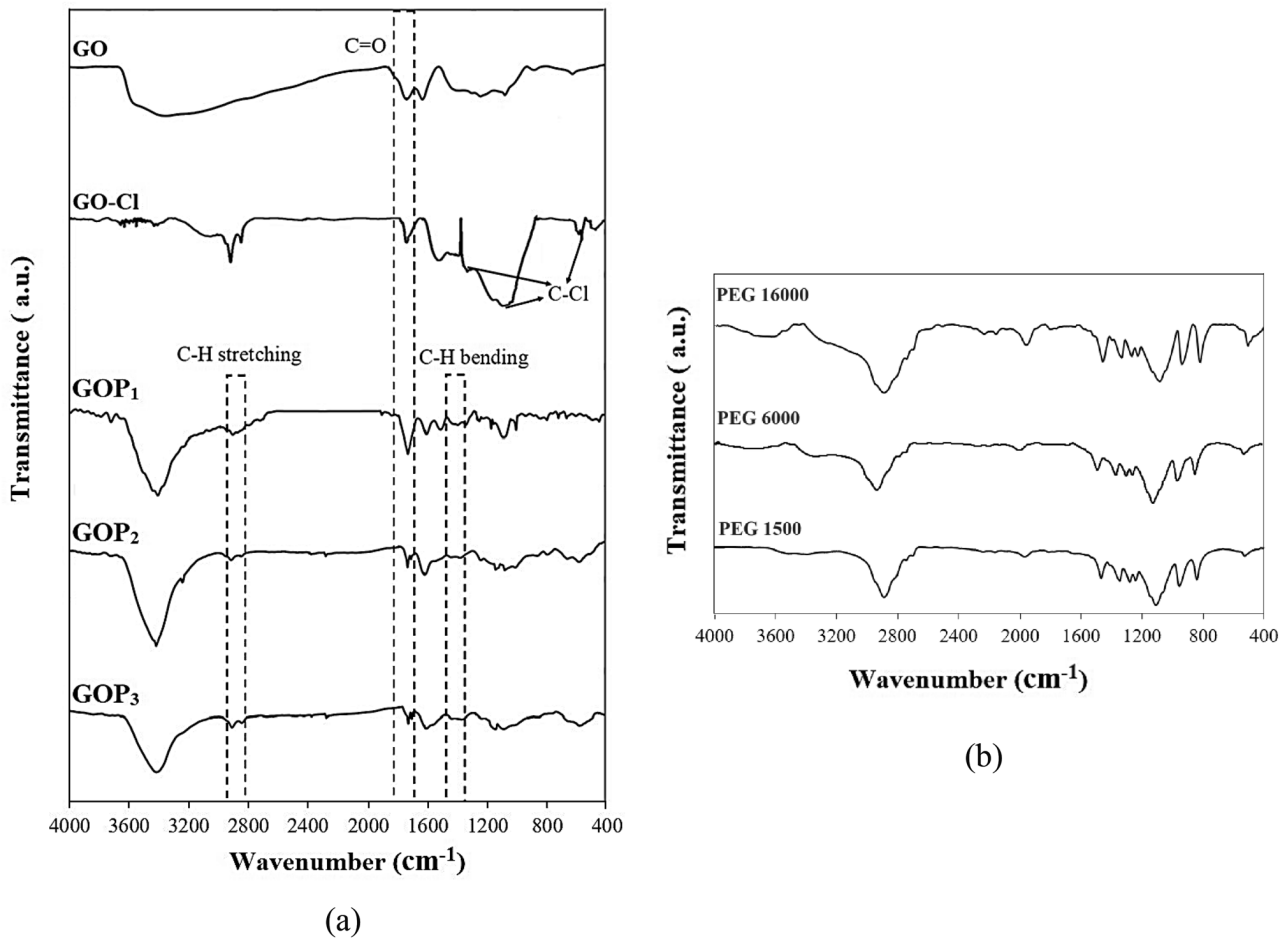
FT-IR spectra of (**a**) GO, GO-Cl and GOP_X_s and (**b**) PEGs with different molecular weights.

Figure 3a exhibits FT-IR spectra of GO, GO-Cl and different GOP_X_ nanosheets. The FT-IR spectra of PEGs with different molecular weights are also shown in Fig. 3b for comparison. The peak associated with the C=O of GO at 1740 cm^-1^ is blue shifted to 1754 cm^−1^ in the spectrum of the GO-Cl representing the acyl chloride formation after the chlorination reaction^26^. The GO-Cl also shows stretching bands at 540 cm^−1^, 1032 cm^−1^ and 1341 cm^−1^ corresponding to C–Cl formation in GO^26^. GOP_X_s show a peak at 1750 cm^−1^ corresponding to the stretching vibrations of C=O bands of their ester groups due to the reaction between hydroxyl groups of PEGs and CO–Cl of GO-Cls^27^. In addition, the peaks at 2923 cm^−1^ and 2853 cm^−1^ can be ascribed to C−H stretching vibrations of CH_2_ groups of PEG. Absorption bands at 1399 cm^−1^ and 1385 cm^−1^ in the spectra of GOP_X_s are representatives of the bending vibrations of the C−H of methylene groups of PEGs grafted on GOs^27^.

**Figure 4:**
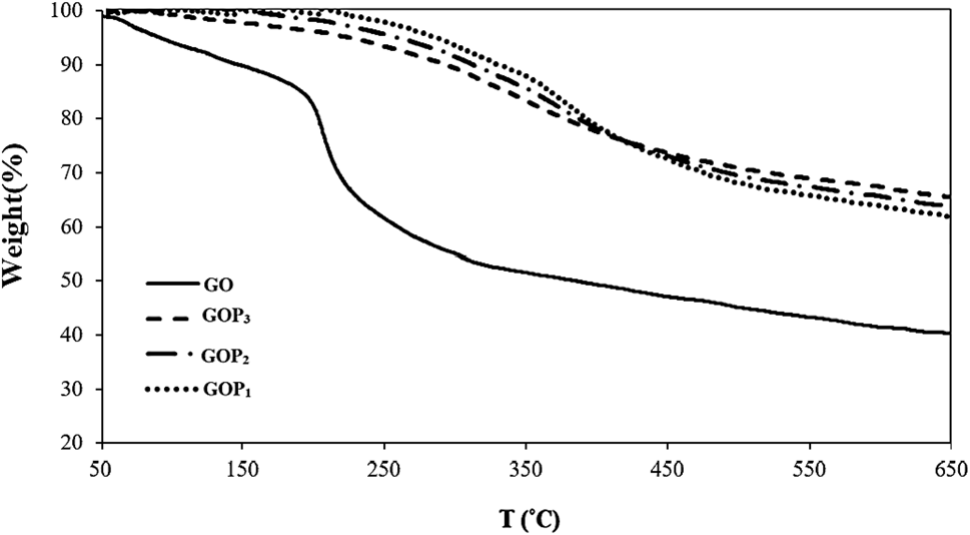
TGA thermograms of GO and GOP_X_s.

Figure 4 shows the TGA curves of the prepared nanosheets. The pristine GO exhibits a significant weight loss (43.2%) in the range of 150−210 °C, due to the decomposition of adsorbed water and unstable oxygen-containing functional groups^28^. For GOP_X_s, with a slower mass loss rate, such a weight loss is not observed at about 200 °C, which may be ascribed to the grafted PEG molecules that cause a reduction in the thermal transport in the nanosheets. This also results in much higher thermal stability of the PEGylated GOs, in comparison with pristine GO, which retains 60–70% of their weight at 650 °C. The thermal stability of the modified GOs follows the order of GOP_1_ > GOP_2_ > GOP_3_, at temperatures lower than 400 °C. However, the reverse holds at temperatures above 400 °C, due to the more thermal stability of PEGs with higher MWs at higher temperatures. Actually, the polar C-O bond is more unstable and breaks easily compared to the nonpolar C–C bond^29^. Below 400 ℃, the weaker C-O bond of the PEG chains starts to degrade. Thus, PEG with a higher MW (16,000 g/mol), which has more C-O bonds than PEG with a lower MW (1500 g/mol) shows lower thermal stability below 400 ℃. On the other hand, above 400 ℃, the higher number of C–C bonds of the higher MW PEG makes it more thermally stable. All the results above reveal that PEGs of different MWs have been successfully grafted onto GOs via ester bonds utilizing SOCl_2_ as an intermediate reagent^30^.

### Characterization of the GOP_X_s/TFN membranes

**Figure 5:**
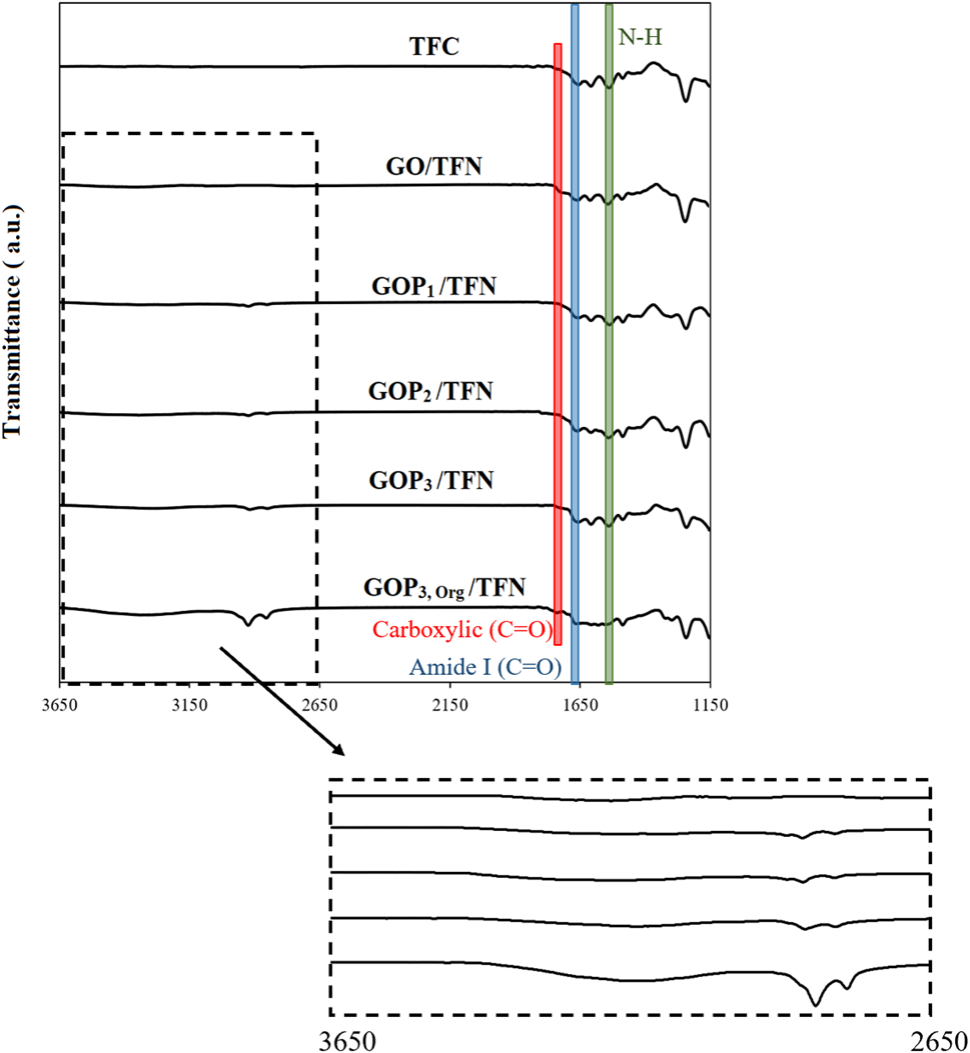
ATR-FTIR spectra of TFC and TFN membranes.

The ATR-IR spectra of the TFC, GO/TFN and GOP_**X**_s/TFN membranes are shown in Fig. 5. All the membranes demonstrate characteristic peaks of the top PA layer at 1660 cm^−1^ and 1547 cm^−1^ due to C=O stretching of -NCHO (amide I) and N–H (amide II), respectively^31^. In GOP_**X**_s/TFN membranes, two peaks at 2918 cm^−1^ and 2849 cm^−1^ can be attributed to the asymmetric and symmetric C–H stretching bands of PEGs, respectively. Moreover, weak broad bands at about 3300 cm^−1^ in the spectra of TFN and membranes are assigned to the stretching vibrations of hydroxyl groups of GO sheets (for GO/TFN) and the combined stretching vibrations of hydroxyl groups of both GO and PEG (for GOP_**X**_s/TFNs)^30–33^. The small peaks at 1710 cm^−1^ in the spectra of TFC and TFN membranes may be ascribed to carboxylic acid C=O stretch resulting from the hydrolysis of some of the acyl chlorides of TMC molecules^20^. This partial hydrolysis of acyl chlorides creates free carboxylic acid groups and impedes complete IP reaction between TMC and MPD monomers thus reducing the cross-linking density of the PA layer. Therefore, the degree of cross-linking of the PA layers can be estimated and compared based on the ATR-FTIR spectra, by calculating the ratio of absorption intensities of amide I (at 1660 cm^−1^) to carboxylic acid C=O stretch (at 1710 cm^−1^) groups: the higher the ratio the larger the cross-linking density^34^. The amide I/carboxylic values for the TFC and TFN membranes are listed in Table 2. As can be seen, the addition of GOPx nanosheets causes a reduction in the crosslinking density of the TFC and TFN membranes. This could be due to the role of the grafted nanosheets with PEG molecules as barriers, which decrease the reaction probability between MPD and TMC monomers and thus increase the possible hydrolysis of acyl chlorides during the IP reaction. In addition, one could expect that annealing of the membranes at 80 °C would result in the evaporation of the residual water and increasing dehydration of carboxylic groups leading to an increase in the PA layer crosslinking density. However, the hydrophilic natures of GO and especially GOPx nanosheets prevent efficient water evaporation, which eventually results in the further hydrolysis of acyl chloride groups of TMC and the reduction of the cross-linked portion of the PA layers of GO/TFN and GOPx/TFNs as compared with that of the PA layer of the TFC membrane.

**Table 2:** Amide I/carboxylic absorption intensity ratio for the TFC and TFNs.

Membranes	TFC	GO/TFN	GOP_1_/TFN	GOP_2_/TFN	GOP_3_/TFN	GOP_3,Org_/TFN
Amide I/carboxylic	9.4	8.1	6.1	6.5	6.6	6.3

**Figure 6:**
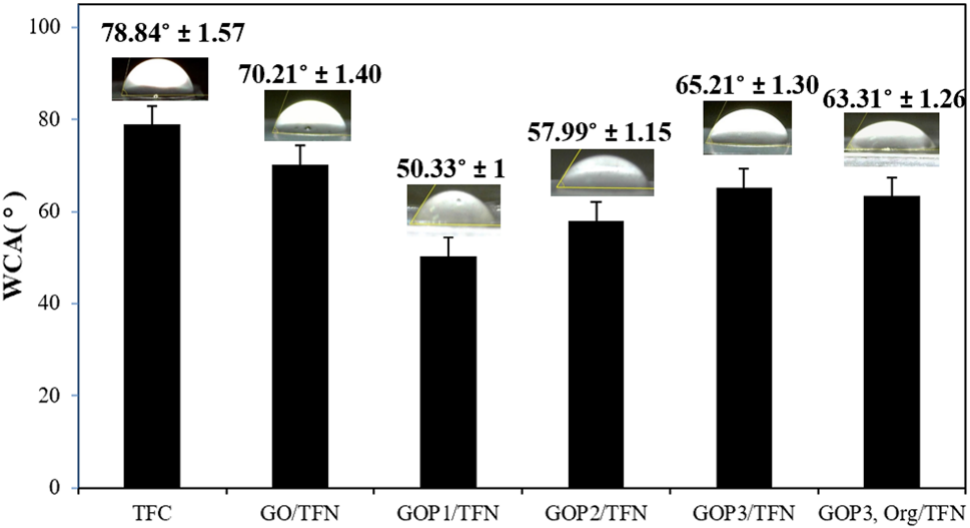
Water contact angles (WCA) of the prepared membranes.

Figure 6 represents the water contact angle (WCA) of the prepared membranes. By introducing the pristine GO, the WCA of the TFC membrane is reduced from 78° to 70°, indicating an improvement in the hydrophilicity of the TFC membrane due to the oxygen-containing functional groups of GO. Furthermore, GOP_X_s/TFN membranes exhibited lower contact angles than TFC and GO/TFN. The hydrophilicity of GOP_x_/TFNs varies in the order of GOP_1_/TFN > GOP_2_/TFN > GOP_3,Org_/TFN > GOP_3_/TFN.

**Figure 7:**
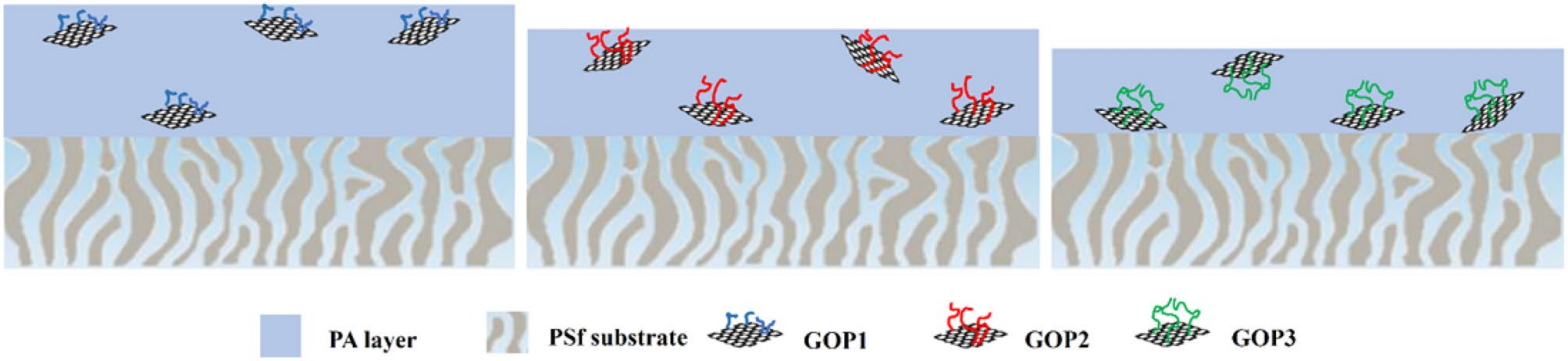
Schematic illustration of how PEG MW affects the distribution of GOP_X_ nanosheets in the PA layer of the GOP_X_/TFN membranes.

The incorporation of GOP_X_ nanosheets increases the hydrophilic groups of the top layers of the TFNs, which form hydrogen bonds with water molecules and hydration layers on the surfaces of the membranes^35^. It is seen that decreasing the MWs of the grafted PEGs increases the hydrophilicity of the GOP_X_s containing TFNs. Dharmaratne et al.^36^ recently showed that PEGs of higher MWs exhibit higher degrees of hydrophilicity. Therefore, GOP_3_ would be more hydrophilic than GOP_2_ and GOP_1_, respectively. When dispersed in the aqueous phase, the more hydrophilic GOP_3_ nanosheets possess more tendency than the other two PEGylated GOs to be in the bulk aqueous phase, while GOP_2_ and particularly, GOP_1_ tend to move partially towards the phase surface. Consequently, as Fig. 7 shows after the interfacial polymerization reaction, more hydrophilic groups are oriented towards the top layer surface of GOP1/TFN and GOP_2_/TFN than the surface of GOP_3_/TFN. This would render GOP_1_/TFN and GOP_2_/TFN surfaces more hydrophilic than the GOP_3_/TFN surface.

**Figure 8:**
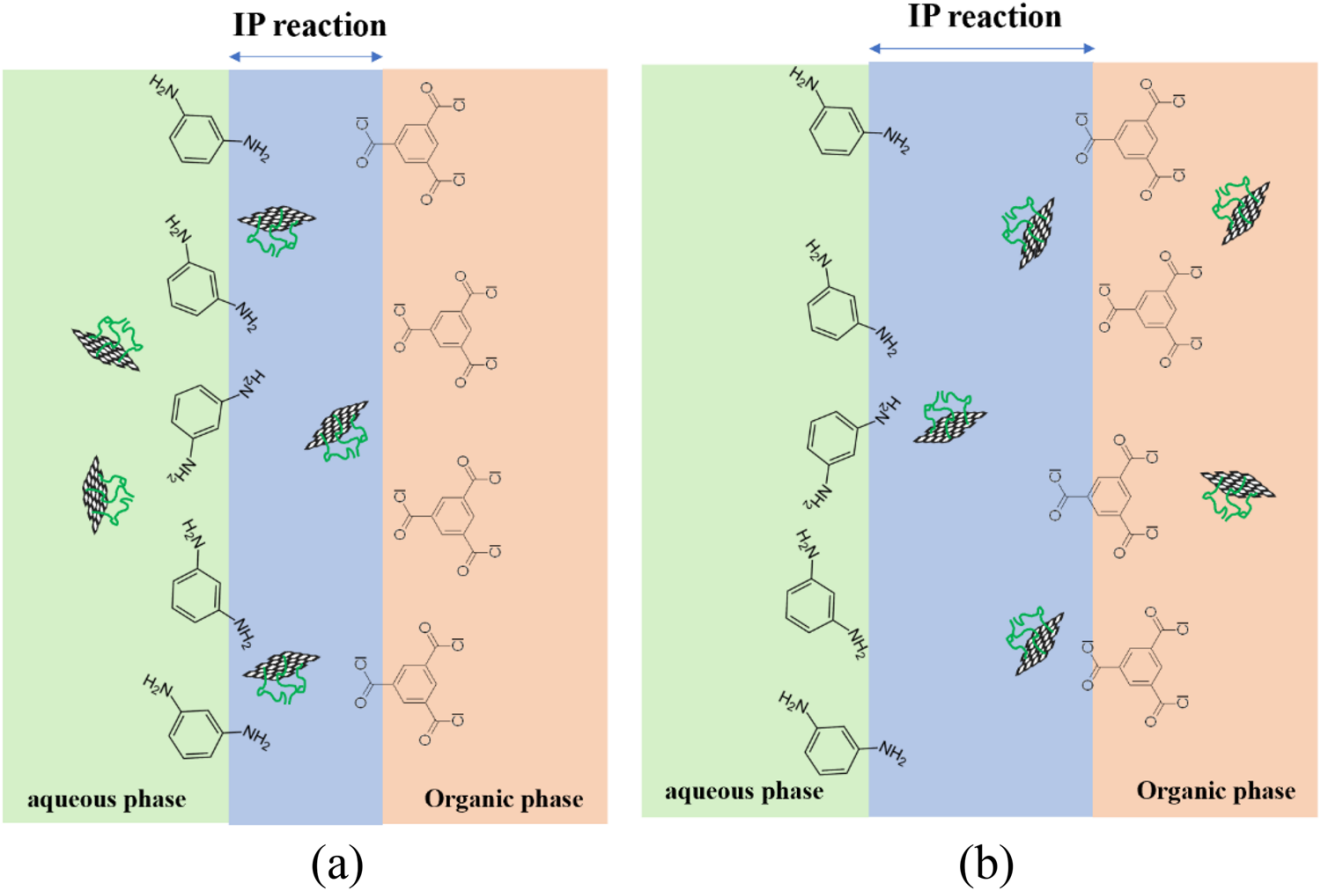
The distribution of GOP_3_ nanosheets in the IP reaction zones of (**a**) GOP_3_/TFN and (**b**) GOP_3,Org_/TFN membranes.

Interestingly, GOP_3,Org_/TFN shows a slightly lower WCA and thus higher surface hydrophilicity than that of the GOP_3_/TFN. This can be ascribed to the diffusion of hydrophilic GOP_3_ nanosheets toward the aqueous phase of IP, during the IP reaction, and their potential trapping on the top layer surface of the GOP_3,Org_/TFN when they have already been dispersed in the organic phase. Figure 8 schematically compares the distribution of GOP_3_ nanosheets in the IP reaction zones of GOP_3_/TFN and GOP_3,Org_/TFN membranes.

**Figure 9:**
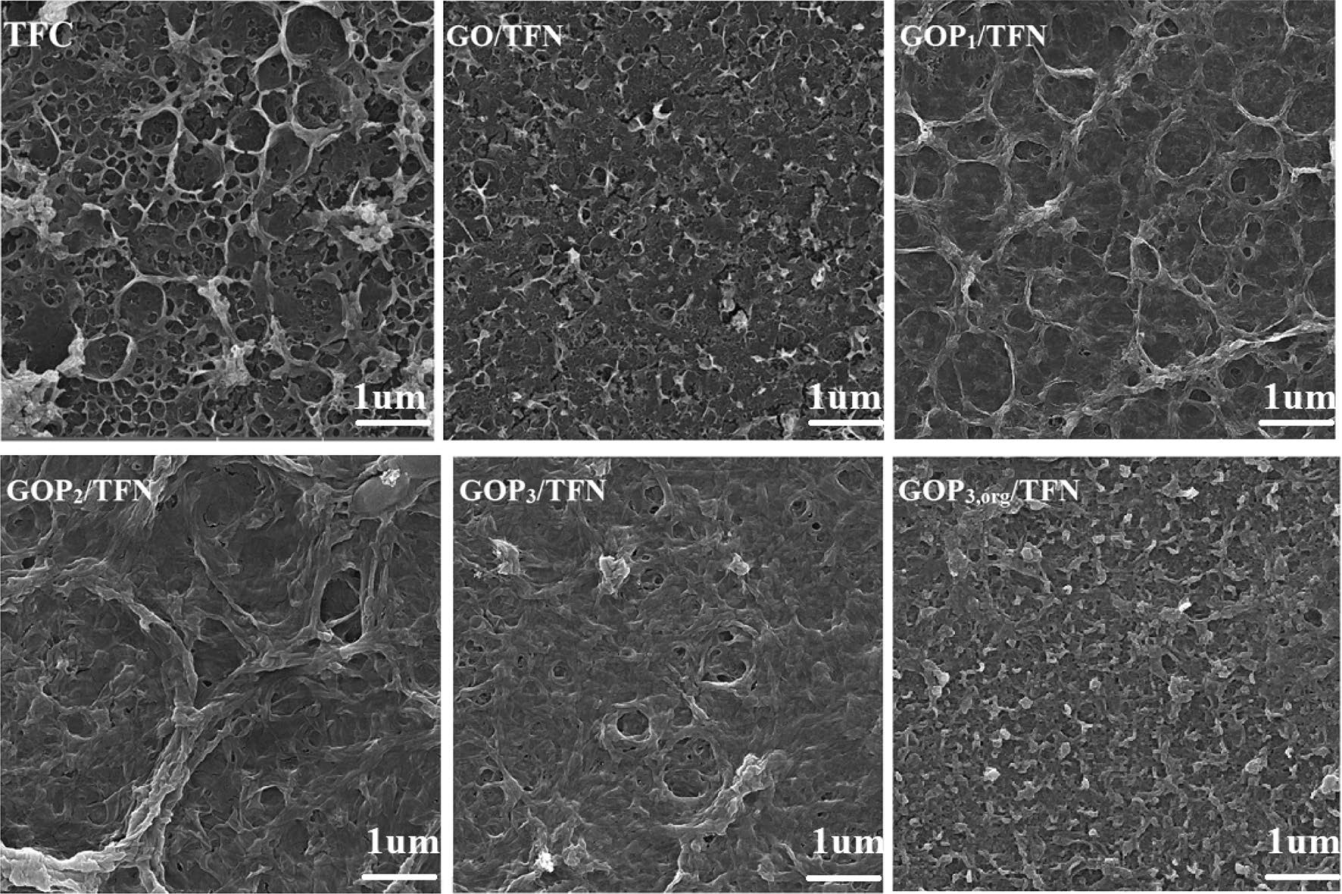
Surface SEM images of the prepared membranes.

**Figure 10:**
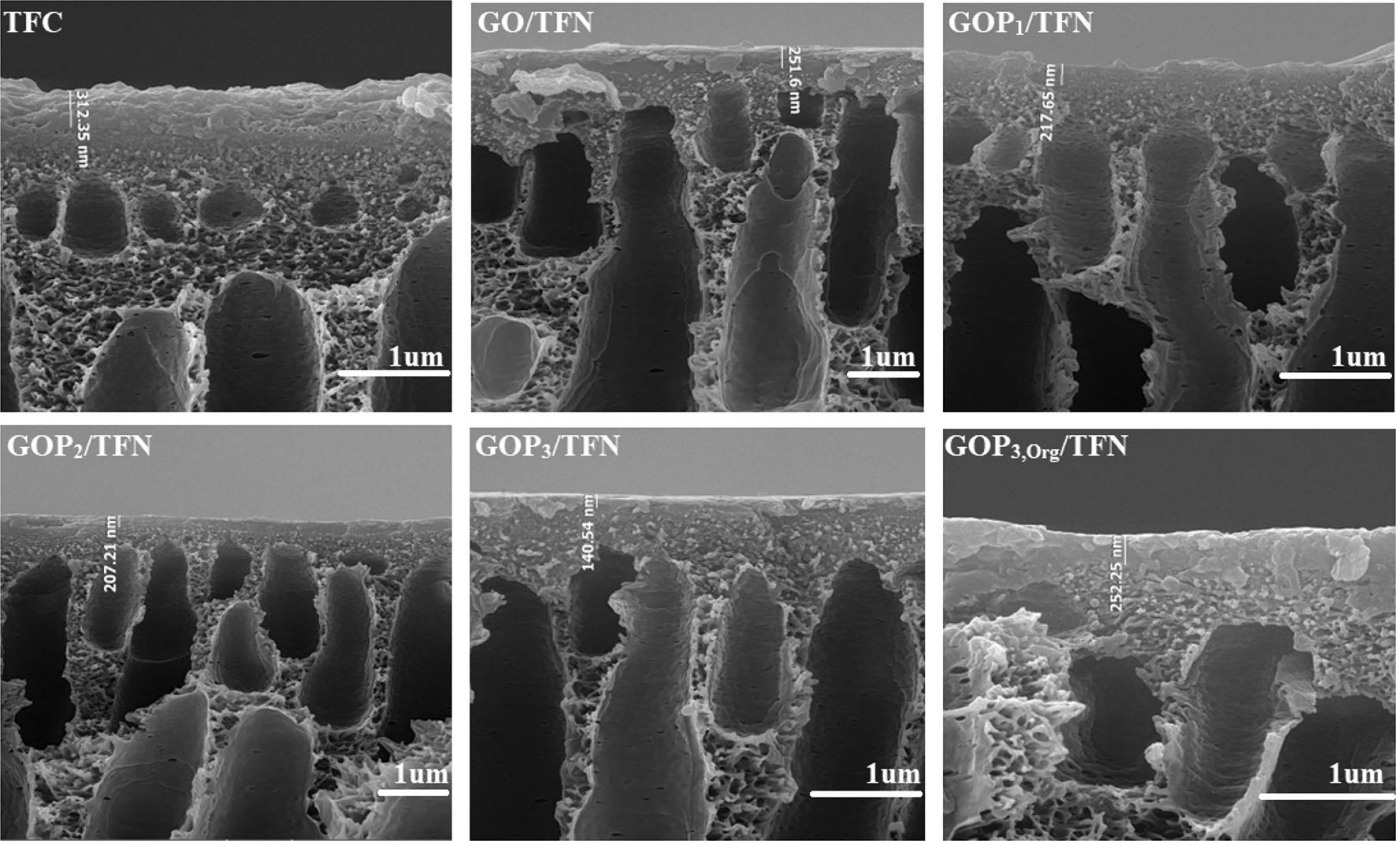
Cross-sectional SEM images of the prepared membranes.

The top surface and cross-sectional SEM images of the fabricated membranes are shown in Figs. 9 and 10, respectively. The TFC membrane exhibits a typical ridge-valley morphology caused by the IP reaction^37^. Meanwhile, the surface morphologies and the top layer thicknesses of the membranes are affected by the incorporation of GO and GOP_X_s in the PA active layer.

**Figure 11:**
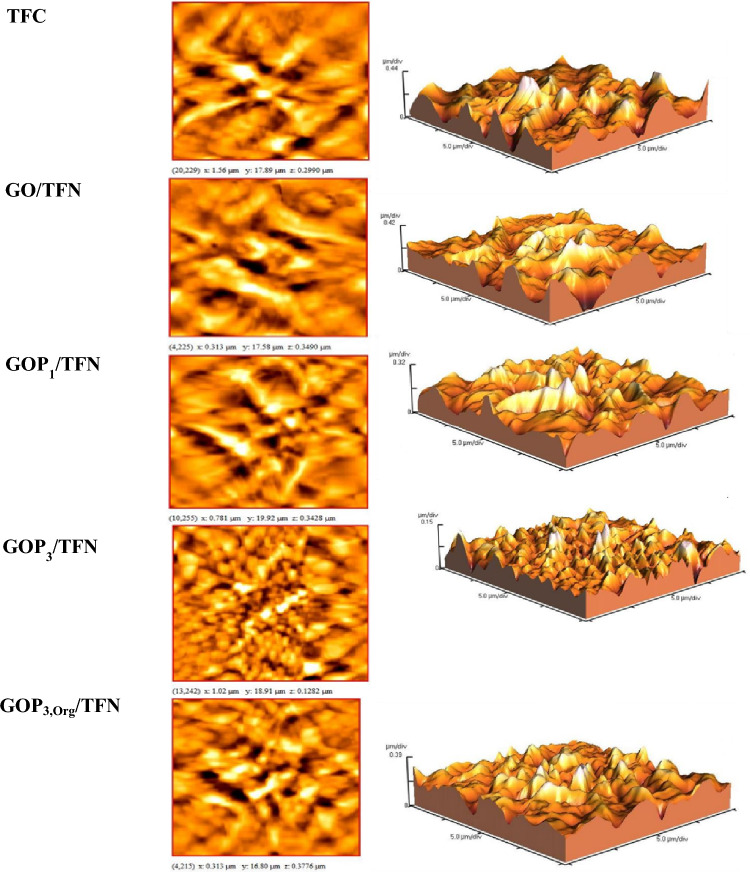
Two- and three-dimensional AFM images of the prepared TFC and TFN membranes.

The layer thicknesses were measured according to the cross-sectional SEM images (Fig. 10) and are reported in Table 3. Moreover, Fig. 11 shows two and three-dimensional AFM images of the TFC and TFN membranes. Table 3 also lists the average roughness (R_a_) and the root means square roughness (Rq) values derived from the AFM images. Introducing GO nanosheets (modified or pristine) into the top PA layer decreases the layer thickness and surface roughness. As the IP reaction is diffusion-controlled in nature, the dispersion of GO and GOP_X_s in the aqueous phase could hinder the diffusion of the aqueous monomer (MPD) toward the organic phase during the reaction due to the creation of a tortuous path for the diffusion of MPD molecules deep into the organic phase. The nanoparticles also increase the viscosity of the aqueous phase. For these two reasons, the formation of ridge-valley structures on the PA surface as well as the PA layer thickness is reduced^8,35^. In addition, altering the IP reaction rate due to the possible interactions of oxygen-containing functional groups of GO and GOP_X_s with MPD could be another reason for the observed morphologies of the TFNs^9,31,33^. Moreover, grafting PEGs onto GOs improves the nanosheet hydrophilicity and their dispersion in the aqueous phase. This can further hinder the diffusion of MPD molecules toward the interface and cause more reductions in the surface roughness and layer thickness of the GOP_X_s/TFN membranes (Table 3).

**Table 3:** Surface roughness parameters and top layer thickness of the prepared membranes.

Membranes	R_a_ (nm)	R_q_ (nm)	The thickness of the top layer(nm)
TFC	70.5 ± 1.5	95.6 ± 3.3	310 ± 3
GO/TFN	56.7 ± 2.2	81.1 ± 3.7	250 ± 2
GOP_1_/TFN	52.6 ± 1.5	72.5 ± 2.2	217 ± 3
GOP_3_/TFN	32.7 ± 1.8	39.6 ± 3.5	140 ± 5
GOP_3,Org_/TFN	56.2 ± 1.7	79.5 ± 4.2	252 ± 5

As expected, PEGs of higher MWs are more effective in increasing the distance between individual nanosheets and improving their dispersions in the aqueous phase of the IP reaction. Moreover, diffusion of MPD is further prevented by longer PEG chains. Consequently, the hydrophilicity and dispersion of the nanosheets would be more enhanced if the PEG MW was increased and therefore, the roughness of the PA surface and thickness of the PA layer of GOP_3_/TFN are lower than those of the GOP_1_/TFN. As Figs. 10 and 11 and Table 3 show dispersing GOP_3_ nanosheets in the organic phase of the IP reaction results in a TFN membrane (GOP_3,Org_/TFN) with higher surface roughness and PA layer thickness than those of the TFN prepared by the IP reaction between GOP_3_**-**containing aqueous phase and organic phase (GOP_3_/TFN). The hindrance effect of GOP_3_ nanosheets on the TMC diffusion in the organic phase is considered to decrease the TMC concentration at the interface and the IP reaction rate. This delays the formation of the diffusion barrier and thus more MPD molecules would diffuse deep into the organic phase, increasing the surface roughness and PA layer thickness^30^.

In addition, hydrophilic GOP_3_ nanosheets in the organic phase increase the miscibility of the organic and aqueous phases of the IP reaction, thereby increasing the reaction zone width, surface roughness and PA layer thickness. Moreover, as compared with the mostly embedded nanosheets inside the PA layer of the GOP_3_/TFN membrane, more GOP_3_ nanosheets would be close to the outer surface of the GOP_3,Org_/TFN membrane. The uneven distribution of the nanosheets on the surface of the GOP_3,Org_/TFN membrane can also lead to a rougher surface than the surface of the GOP_3_/TFN one^38^.

### RO performance of the membranes

**Figure 12:**
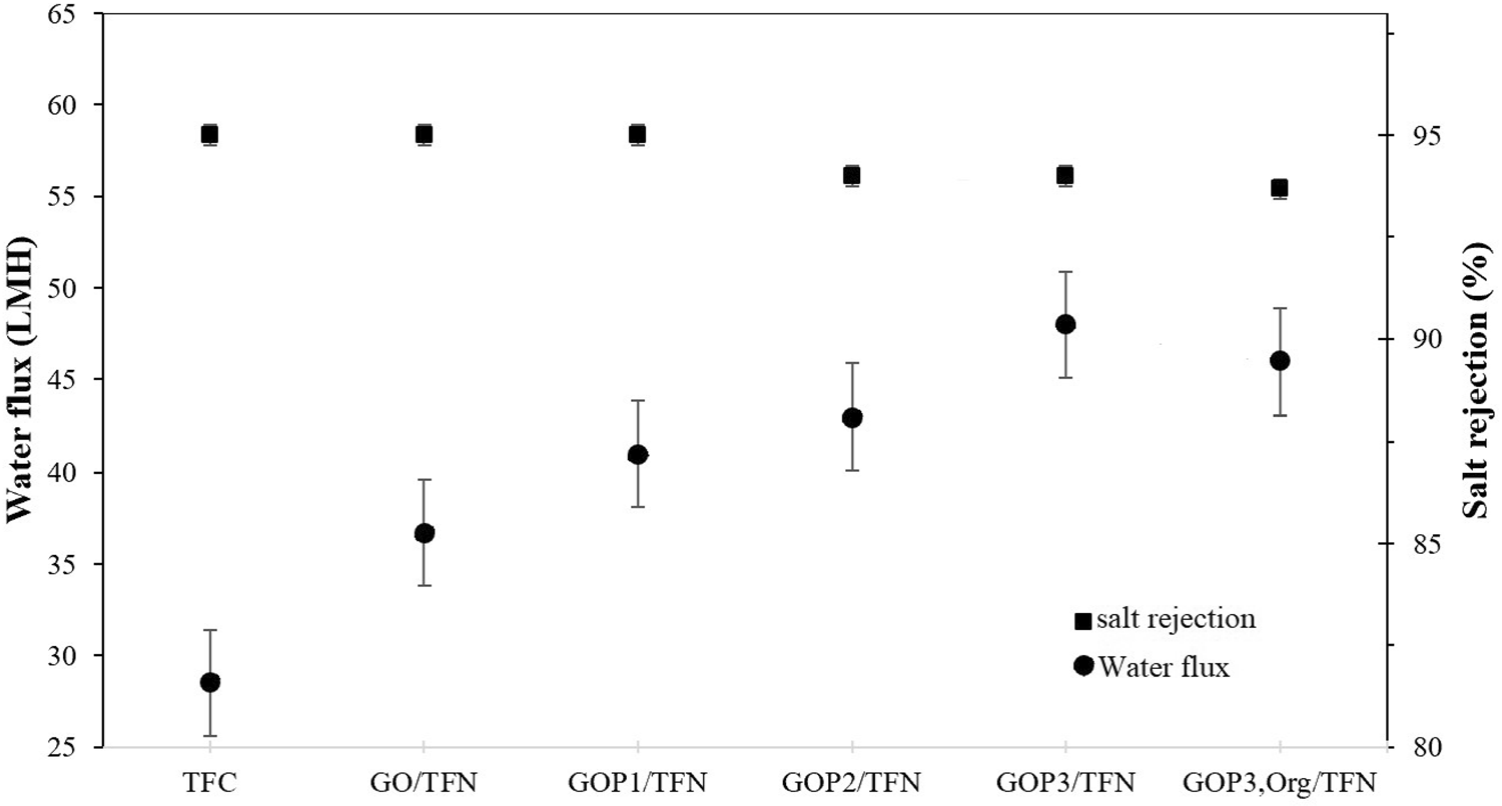
Water flux and salt rejection of the prepared membranes.

Figure 12 shows the water flux and NaCl rejection of the prepared membranes. The bare TFC membrane shows the lowest water flux among the membranes. The water flux of the TFC membrane, 28.5 LMH, is enhanced by 28.8%, 44%, 51%, 68.4% and 61.4% in GO/TFN, GOP_1_/TFN, GOP_2_/TFN, GOP_3_/TFN and GOP_3,Org_/TFN, respectively (Fig. 12 and Table 4). On the other hand, the salt rejection of the TFC membrane remained almost constant, more than 94%, with the addition of GO and GOP_X_s in the PA layer. This indicates that although the improvements in water flux of GO-containing membranes may be partly due to the decrease in their crosslinking density compared to that of the bare TFC (Table 2), the platelet nature of GO nanosheets and their role in creating tortuous paths for salt molecules retain the membrane's selectivity at a high level. Another possible reason for increasing the water flux by embedding the GOPx nanosheets in the PA layers could be the introduction of hydrophilic groups on the surface and in the bulk of the polyamide layer of the GOPx-containing TFN membranes as confirmed by contact angle measurements (Fig. 6). The GOPx nanosheets on the surface and in the bulk of the polyamide layer could help dissolve water molecules on the membrane surface and improve the diffusion rate of water molecules passing through the TFN membrane, respectively^8^.

**Table 4:** RO performance of the TFC and TFN membranes.

Membranes	Salt rejection (%)	Water flux (L/M^2^H)	Permeance (L/M^2^.H.bar)	Slope of flux-time profiles
TFC	95 ± 0.95	28.5 ± 0.57	1.8 ± 0.03	−0.0607
GO/TFN	95 ± 0.95	36.7 ± 0.73	2.3 ± 0.04	−0.0503
GOP_1_/TFN	95 ± 0.95	41.0 ± 0.82	2.5 ± 0.05	−0.0313
GOP_2_/TFN	94 ± 0.94	43.0 ± 0.86	2.7 ± 0.05	−0.0319
GOP_3_/TFN	94 ± 0.94	48.0 ± 0.96	3.0 ± 0.06	−0.0264
GOP_3,Org_/TFN	94 ± 0.94	46.0 ± 0.92	2.8 ± 0.05	−0.0336

Furthermore, as discussed in the previous section, the embedding of GO and GOP_X_s significantly reduced the thickness of the top layer of TFN membranes decreasing the resistance to mass transfer and increasing the water flux. Moreover, the presence of hydrophilic PEGs with different MWs on GOP_X_s nanosheets increases the d-spacing between GO nanosheets and creates water-attractive channels through which water can flow even more easily across the membranes^33^.

Figure 12 shows that the increase in the MW of PEGs grafted on the GOs enhances the water flux of the membranes, i.e., the flux of GOP_X_/TFNs varies in the order GOP_3_/TFN > GOP_2_/TFN > GOP_1_/TFN. As Table 3 shows, increasing the PEG MW was accompanied by decreases in the PA layer thicknesses of GOP_X_/TFNs membranes. This can be the main reason behind the observed increase in the water flux of TFNs containing GOs grafted with PEGs of higher MW. However, the above trend of the water flux of GOP_X_/TFNs membranes seems to be contradictory with the contact angle results (Fig. 6), as one expected that a TFN membrane with a lower contact angle and higher surface hydrophilicity should exhibit higher water flux. This discrepancy can be resolved by the fact that GOs grafted with PEGs of higher MW further enhance the bulk hydrophilicity, as discussed earlier, and provide preferential paths for water molecules across the membranes. Consequently, combined effects of lower PA layer thickness and higher PA bulk hydrophilicity contributed to the observed enhancements of the water fluxes of GOP_X_/TFNs membranes. It is also seen from Fig. 12 that the water flux of the GOP_3,Org_/TFN membrane is lower than that of the GOP_3_/TFN membrane. This can be ascribed to the lower PA bulk hydrophilicity and higher thickness of the PA selective layer of the former membrane, as deduced from Fig. 6 and Table 3, respectively, leading to higher resistance against water flow through the membrane.

**Figure 13:**
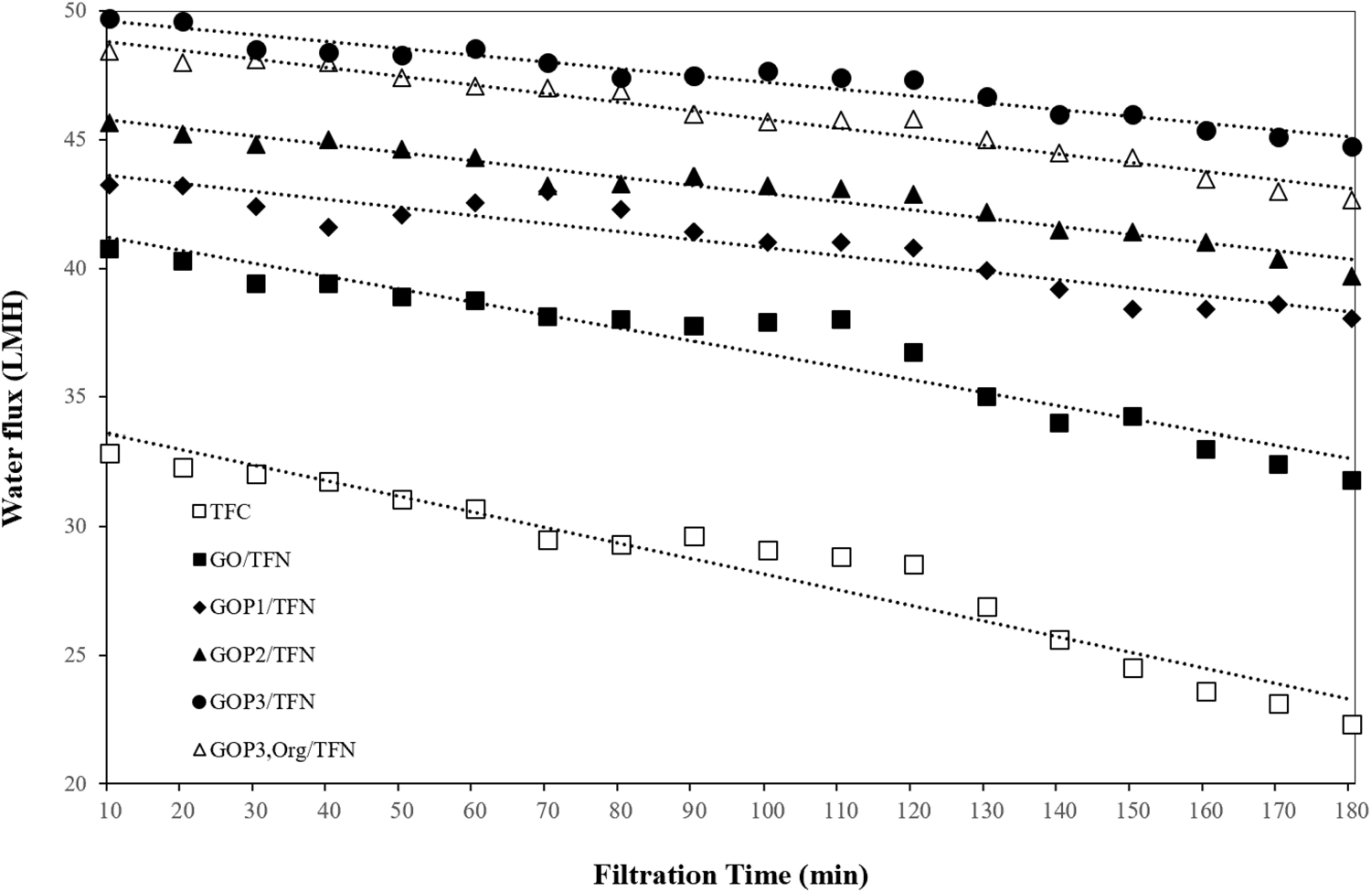
Water flux-time profiles of the prepared membranes (dotted lines indicate trend lines).

Time dependency of water flux of membranes is an important indicator of the membranes' propensity to fouling^33^. According to Fig. 13, the fluxes of all the TFC and TFN membranes are decreased with the time passed from the filtration process which indicates the occurrence of varying degrees of fouling during the water filtration. As listed in Table 4, the absolute value of the trend line slope of the flux-time profiles of the membranes increases in the order of GOP_3_/TFN < GOP_1_/TFN ≅ GOP_2_/TFN < GOP_3,Org_/TFN < GO/TFN < TFC. In other words, bare TFC with the highest permeance reduction rate among the membranes would be more prone to fouling, compared to GOPx/TFN and GOP_3,Org_/TFN membranes, which show the lowest flux reduction rate and thus the greatest anti-fouling properties. These potential anti-fouling properties can be attributed to the high surface hydrophilicity and low surface roughness (Table 4) of GOPx/TFN and GO/TFN membranes, which would lead to the deposition of a lesser amount of hydrophobic foulants on the membrane’s surfaces.

Table 5 compares the water permeance and NaCl rejection of GOP_X_-containing membranes with those of other GO-containing TFNs in the literature. As can be seen, GOP_X_/TFN membranes outperform most of the lab-fabricated relevant TFN RO membranes in terms of water permeance, although the use of hybrid nanoparticles, e.g., TiO_2_-GO nanoparticles, led to a high water permeance of 6.2 LMH/bar^44^. Furthermore, GOP_X_/TFNs show acceptable NaCl rejection, which is higher than those of commercial membranes, e.g. SWHR (FilmTec Corp. Edina, MN) with a NaCl rejection of 92%^45^.

**Table 5:** Comparison of the water permeance and NaCl rejection of GOPxs-containing membranes with those of other GO-containing TFNs in the literature.

Nanoparticles	Loading (wt%)	IP phase in which nanosheets were dispersed	Water permeance (LMH/bar)	NaCl rejection (%)	Reference
GO	0.12	Aqueous	0.22	96.78	^32^
GO	0.01	Aqueous	1.97	97	^39^
GO	0.0053	Organic	2.30	97	^40^
GO	0.0038	Aqueous	1.07	99.4	^17^
GO	0.10	Organic	2.87	93.8	^41^
GO	1	Coated on PA	0.90	96.40	^42^
GOQD	0.1	Aqueous	2.34	98.8	^43^
GO-PSBMA	0.3	Aqueous	0.42	90	^9^
Aminophenol-GO	0.005	Aqueous	1.57	99.7	^6^
ZGO	0.02	Aqueous	1.46	94.8	^8^
TiO_2_-GO	0.001	Aqueous	6.2	97	^44^
GO-ZnO	0.02	Aqueous	1.57	96.3	^46^
GO@CS	0.01	Aqueous	1.57	99.1	^47^
GOP_1_	0.004	Aqueous	2.50	94	This work
GOP_2_	0.004	Aqueous	2.70	94	This work
GOP_3_	0.004	Aqueous	3.00	94	This work
GOP_3_	0.004	Organic	2.88	94	This work

## Conclusions

This study aimed at exploiting simultaneously the advantages of both GO nanosheets and PEG polymer in the PA layer of TFC RO membranes. GO nanosheets were grafted with PEGs of different MWs (GOP_X_) via a facile grafting approach and successfully incorporated in the thin PA layer of TFC membranes. GOP_X_ nanosheets improved water flux while maintained high salt rejection of the bare TFC and pristine GO-containing TFN. Among GOP_X_s, GOP_3_ nanosheets endowed TFNs with the highest PA bulk hydrophilicity, the lowest PA layer thickness and surface roughness and thus the best performance in the RO process. Moreover, it was shown that the TFN prepared by the addition of GOP_3_ nanosheets to the aqueous phase of the IP reaction possessed higher water flux than the TFN prepared by adding the nanosheets to the organic phase. Finally, the high antifouling potential of GOP_X_/TFN membranes was confirmed by analyzing and comparing the slopes of the water flux-time profiles of the prepared membranes.

## Data Availability

The datasets used and/or analyzed in the current study are available from the corresponding author on reasonable request.
